# The impact of vitamin D supplementation on VDR gene expression and body composition in monozygotic twins: randomized controlled trial

**DOI:** 10.1038/s41598-020-69128-2

**Published:** 2020-07-20

**Authors:** Jeane Franco Pires Medeiros, Michelle Vasconcelos de Oliveira Borges, Aline Alves Soares, Jessica Cavalcante dos Santos, Ana Beatriz Bezerra de Oliveira, Conceição Horrana Belo da Costa, Marina Sampaio Cruz, Raul Hernandes Bortolin, Renata Caroline Costa de Freitas, Paulo Moreira Silva Dantas, Mario Hiroyuki Hirata, Vivian Nogueira Silbiger, André Ducati Luchessi

**Affiliations:** 1 Department of Health Sciences, Federal University of Rio Grande Do Norte, Av. General Cordeiro de Farias, S/N – Petrópolis, Natal, RN 59012-570 Brazil; 2 Department of Pharmaceutical Sciences, Faculty of Pharmacy, Federal University of Rio Grande Do Norte, Natal, RN Brazil; 3 Graduate Program in Pharmaceutical Sciences, Faculty of Pharmacy, Federal University of Rio Grande Do Norte, Natal, RN Brazil; 4 Department of Clinical and Toxicological Analyses, School of Pharmaceutical Sciences, University of Sao Paulo, Sao Paulo, SP Brazil; 5 Department of Clinical and Toxicological Analyses, Federal University of Rio Grande Do Norte, Natal, RN Brazil

**Keywords:** Nutrition, Quality of life, Molecular biology, Diseases, Health care, RNA

## Abstract

Vitamin D supplementation is widely used. However, there is no consensus on the use and dosage of this supplement and the existing recommendations arise from studies based on the benefits that this nutrient can facilitate in bones. In addition, individual genetics can influence the response to supplementation, therefore, research involving monozygotic twins aims to reduce these differences in phenotypic responses. The objective of this randomised controlled study is to examine the effect of vitamin D supplementation on body composition and the expression of the vitamin D receptor (*VDR*) mRNA. An intervention was performed through supplementation with cholecalciferol at the concentration of 2000 IU in 90 healthy adult monozygotic twins (male or female pairs) for 2 months. The findings showed that serum vitamin D concentration increased by 65% and *VDR* gene expression sixty times (*p* = 0.001). Changes in body composition parameters were observed regarding body fat and lean mass. Our results indicate that an increase in serum vitamin D concentration may have potential therapeutic implications.

## Introduction

Supplementation with vitamin D (cholecalciferol) has become a widely used practice, since a relationship has been demonstrated of low levels of this nutrient with the increased risk of cardiovascular diseases, the recurrence of diseases, and mortality^1,2,3^. However, as there is no consensus on sufficient serum levels of vitamin D, taking into account its non-skeletal functions, it is necessary to assess whether the increase in this nutrient in individuals with 25-hydroxyvitamin D (25 (OH) D) levels above the current cut-off point, generates any health benefits^4^.

Vitamin D is involved in several non-skeletal functions, such as cell regulation, differentiation, and growth^3^, and adaptive and innate immunity control^5^, as well as being associated with inflammatory markers^6^, since the vitamin D receptor (*VDR*) is expressed in almost all human cells^7^. The activity of this nutrient in the human organism involves its binding with *VDR*^8^, whose expression is modulated by the blood levels of the 1,25diidroxi-vitamin D (1,25 (OH) 2D)^9^ and genetic variants^10^.

It is believed that there is a negative correlation between the concentration of 25 (OH) D with the body mass index (BMI) and percentage of fat mass^11^. However, few studies have comprehensively evaluated the effect of increasing vitamin D concentration on body composition^1^, and no studies have analyzed the associated gene expression. In addition, clinical studies with monozygotic twins (MZ) are molecular models that allow evaluation of phenotypic aspects with minor interference related to individual genetic variability^12,13^, since MZ twins come from the same zygote^14^. Thus, a gene expression study of vitamin D supplementation in this population is an innovative idea, since individual genetic variations influence serum levels of this micronutrient^15,16^.

The aim of this study is to evaluate the effect of cholecalciferol supplementation on genic expression of the genes *VDR,TNFa*, and *PPARa* and body composition in MZ twins.

## Methods

### Study design and participants

This is a randomized clinical trial in pairs of MZ twins. This was a blind study, where each individual received an identification code, so that the researchers responsible for collecting data, carrying out tests, and analyzing the results did not know who had received the supplement. Twins who participated in the 1st Rio Grande do Norte Twin Festival were included in the study. The zygosity of the twins was determined by means of the zygosity questionnaire^17^. Eligibility criteria included pairs of twins of the same sex, from 18 to 45 years of age (to decrease the age gradient), twins who did not use vitamin supplements, with no diagnosis of chronic non-communicable diseases, and non-smokers. Individuals who were users of illicit drugs, those using supplements containing vitamin D including pregnant women, nursing mothers, and patients with metallic implants and pacemakers, that make it impossible to perform the Dual Energy X-ray absorptiometry test were excluded from the study. No amendments were made to the protocol of the research after the initiation of the trial and follow-up.

This study was approved by the Research Ethics Committee of the Federal University of Rio Grande do Norte (UFRN), protocol number 1.385.218, CAAE 51186615.5.0000.5292, which follows the resolutions of the National Research Ethics Commission of Brazil, in accordance with the principles of the Declaration of Helsinki. It was also approved by the Brazilian Registry of Clinical Trials under registration number RBR-3qy2f2 (date of registration 28/03/2016). All experiments were performed in accordance with relevant guidelines and regulations.

All participants were informed of the research objectives by responsible researchers and informed consent was obtained from each one. Their personal information was kept confidential.

### Randomization

**Figure 1:**
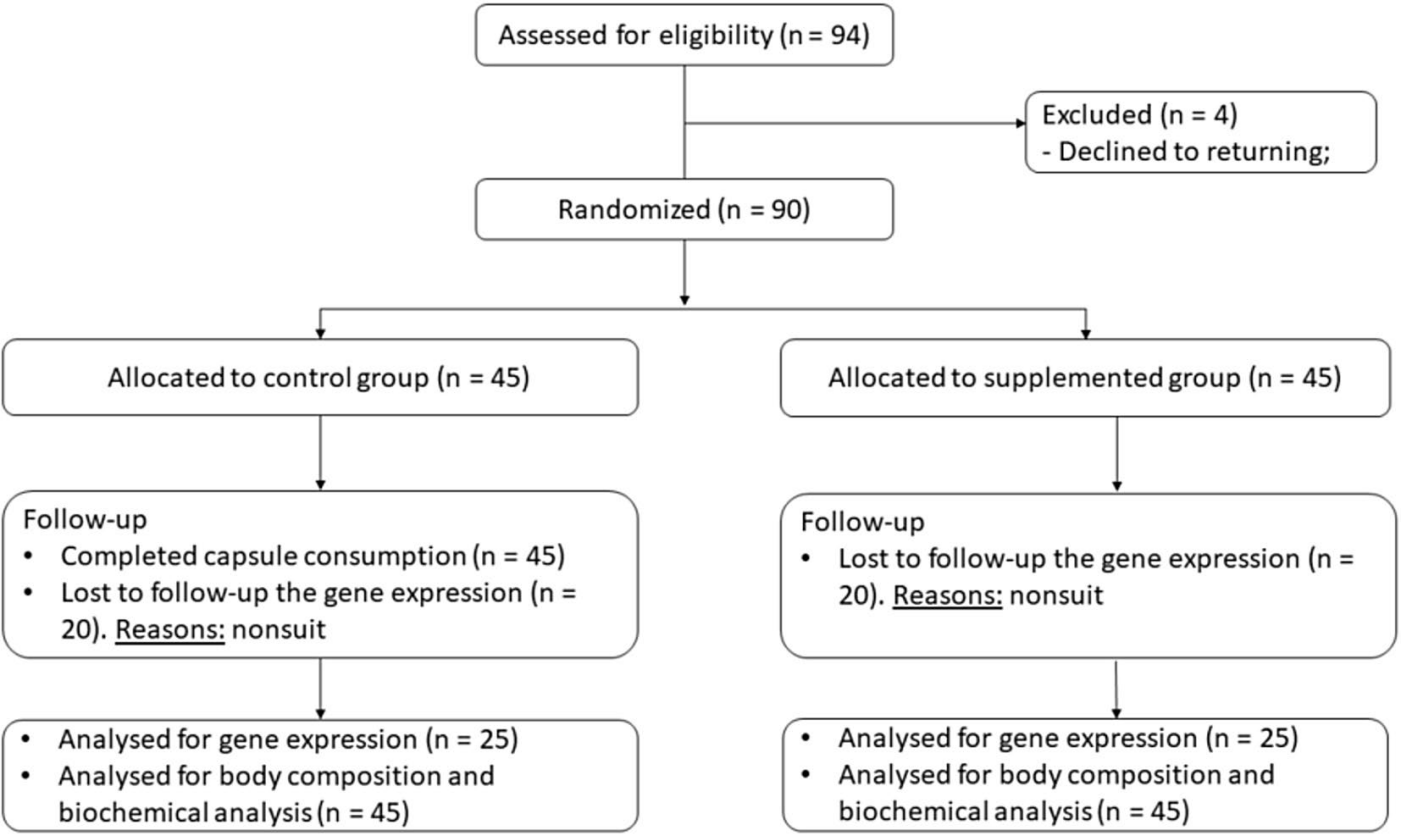
CONSORT diagram of participants of the randomized trial. Flow diagram of the study design. Dropouts from the study occurred due to giving up on returning for collection.

Randomization occurred by lot, performed centrally between 06 June 2016 and 21 December 2018 (at the Laboratory of movement at the Federal University of Rio Grande of Norte) using computer generated sequences stratified for twin 1 and twin 2, where each pair of twins received a numeric code and was separated, with one twin allocated to the control group (CG, without intervention) and the other to the supplemented group (SG, with intervention). The researcher responsible for the registration of the participants and for generating the randomisation and allocation of the assigned intervention were unique and different. The last follow-up occurred on February 21, 2019. In total, 94 eligible twins were recruited, and after being given information about the study, 90 individuals (73% women and 27% men) agreed to participate through written consent. There was sample loss only for analysis of gene expression of 40 individuals, since they did not return for collection regarding this analysis. However, to obtain a statistical power of 0.958, using the values of Mean and Standard Deviation, at least 25 individuals are required before supplementation and 25 individuals after supplementation in these analyzes. In the other variables evaluated the 90 individuals remained from beginning to end. The study complied with the Consolidated Standards of Reporting Trials—CONSORT (Fig. 1).

The twins included in the SG received 60 capsules containing cholecalciferol with a concentration of 2000 IU each, and were instructed to ingest one capsule per day for 60 days. This concentration does not exceed the maximum tolerable daily intake limit (UL), which is 4,000 IU/day^18^, in addition to being considered a dose for maintaining adequate levels of vitamin D in individuals after treatment for deficiency of this nutrient^4^.

At the beginning of the research, the primary endpoint was considered as the variation in the serum concentration of 25 (OH) D after 60 days of supplementation with cholecalciferol. Our secondary endpoint was the variation in gene expression and body composition after 60 days of cholecalciferol supplementation.

During the intervention period, the twins maintained their usual routine, without dietary, sports, or lifestyle changes. After 30 days of inclusion in the study, participants from both groups were interviewed by a phone call to confirm appropriate capsule intake and for routine maintenance. All participants received a follow-up form to daily record when they ingested each capsule. Sixty days after the start of supplementation, these forms were returned to the researchers.

### Body composition

Body composition was estimated by Dual Energy X-ray absorptiometry, performed using Lunar Prodigy Densitometer equipment, Model NRL 41990 (GE Lunar, Madison, WI, USA). The body mass index (BMI), total body fat and by body part, total lean mass and by body part, and bone mineral density (BMC) were measured on the day of inclusion in the study (T0, time 0) and 60 days after the first analysis (T60, time 60) in all participants in the CG and SG groups.

To calculate the BMI, height and body weight were measured using a stadiometer.

### Sun exposure and skin phototype

The assessment of sun exposure was obtained through a score corresponding to the 7 days prior to the date of the bone densitometry evaluation^19^. The use of sunscreen was also recorded. The skin phototype was classified according to a questionnaire proposed by Fitzpatrick^20^.

### Biochemical and vitamin D measurement

Peripheral blood samples were obtained after a 12-h fast in all participants in the CG and SG groups at T0 and T60. Glucose levels, total cholesterol, high-density lipoprotein (HDL), triglycerides, alanine aminotransferase (ALT), aspartate aminotransferase (AST), total proteins, creatinine K, and uric acid were measured using Labtest kits (Lagoa Santa, Brazil) and Labmax Plenno equipment (Labtest, Lagoa Santa, Brazil).

The serum concentration of 25-hydroxyvitamin D (25 (OH) D) was measured by chemiluminescence (Access 2, Beckman Coulter, United States). Values below 20 ng/mL were considered as deficiency and values between 20 and 29 ng/mL as insufficiency^4^.

### RNA extraction

4,0 mL of peripheral blood samples were collected from patients in tubes containing EDTA and leukocytes were isolated by centrifugation (1,340× *g*, 15 min) for gene expression analysis. Total RNA was extracted from leukocytes conserved in RNA*later* stabilization solution (Invitrogen, California, USA) using Trizol reagent (Thermo Fisher, Waltham, MA, USA) according to the manufacturer’s protocol**.** RNA quantification and purity were assessed by spectrophotometry using a Nanodrop ND-1000. RNA samples were stored at − 80 °C until RT-qPCR analysis.

### RNA expression by RT-qPCR

cDNA was synthesized with 50 μg of total RNA using the High-Capacity cDNA Reverse Transcription Kit (Thermo Fisher, Waltham, MA, USA), in a MyCycler Thermal Cycler (Bio-Rad, Philadelphia, PA, USA), according to the manufacturer’s protocol, and stored at − 20 °C.

RT-qPCR was carried out in 384-well plates using specific primers for Vitamin D Receptor Proteins (*VDR),* Peroxisome Proliferator Activated Receptor Alpha *(PPARa),* Tumor Necrosis Factor Alpha (*TNFa),* Actin Beta *(ACTB)*and the QuantiTect SYBR Green PCR Kit (Qiagen GmbH, Hilden, Germany) (Supplementary Table 2 for sequence).

The qPCR assays were performed in a Quant Studio 12 K Flex Real-Time PCR System (Applied Biosystems, Forest City, USA). mRNAs with Ct-values > 35 were excluded from the statistical analysis. Glyceraldehyde-3-Phosphate Dehydrogenase (*GAPDH)*, 18S rDNA, and *ACTB* were used as reference genes. According to the NormFinder algorithm, ACTB was the most stable gene under our experimental conditions, and thus it was used as the endogenous control. Relative expression was calculated by the 2^−ΔΔCt^ method^21^, using the non-supplemented group as the control.

### Statistical analysis

Distribution of variables was analyzed using the Kolmogorov–Smirnov test. Medians and quartiles are used to describe non-normally distributed data and absolute number and percentages to describe categorical data. The Mann–Whitney test was utilized to analyze the difference in independent group samples and the Wilcoxon test for dependent samples to verify intragroup differences. For categorical samples, the Chi-Square test was performed.

Statistical analysis was performed using the statistical software IBM SPSS version 20.0 for Windows (SPSS Inc., Chicago, IL, USA). All differences were considered significant when *p* ≤ 0.05.

## Results

### Baseline characteristics

In total, 90 twins completed the study (see CONSORT diagram, Fig. 1). The rate of adherence and fidelity to capsule consumption was 100% in the SG (n = 45). Participants who ingested the 2000 IU cholecalciferol supplement reported no side effects. Sample collections took place in the years 2018 and 2019.

Socioeconomic data, including schooling and family income, were not significantly different between the CG and SG (Table 1). The most prevalent phototype of skin in this study was phototype 3 (light brunette skin, moderately burning and tanning, normal sensitivity to the sun). The sun exposure score was 16 at time 0 and time 60 in both groups. Over 70% of the total subjects did not use sunscreen daily, and those who did restricted use to the face.

**Table 1:** General characteristics of the twins in the study.

	CG	SG	*p* value
	(n = 45)	(n = 45)	
Age, years	24.0 (21.0–27.0)	24.0 (21.0–27.0)	
**Education level** ***n***, **(%)**			
Incomplete secondary education	1.0 (2.2)	1.0 (2.2)	
Complete secondary education	8.0 (17.8)	10.0 (22.2)	
Incomplete higher education	12.0 (26.7)	18.0 (40.0)	
Complete higher education	14.0 (31.1)	12.0 (26.7)	
Postgraduate	10.0 (22.2)	4.0 (8.9)	0.386
**Family income level** ***n***, **(%)**			
< 1 Minimum wage	5.0 (11.1)	0.0 (0.0)	
> 3 < 5 Minimum wages	12.0 (26.7)	11.0 (24.4)	
> 5 < 7 Minimum wages	9.0 (20.0)	3.0 (6.7)	
> 7 < 10 Minimum wages	7.0 (15.6)	7.0 (15.6)	
> 10 < 20 Minimum wages	3.0 (6.7)	9.0 (20.0)	
> 20 Minimum wages	4.0 (8.9)	4.0 (8.9)	
Does not know	3.0 (6.7)	7.0 (15.6)	
Did not answer	2.0 (4.4)	4.0 (8.9)	0.065
Wears sunscreen *n*, (%)	10.0 (22.2)	11.0 (24.4)	
Does not wear sunscreen *n*, (%)	35.0 (77.8)	34.0 (75.6)	0.758

Categorical variables are shown as number (percentage) and compared by chi-square test. Continuous variables are shown as median (percentile 25–percentile 75) and compared by Wilcoxon test. CG, Control group; SG, Supplemented group; BMI, body mass index; BMC, bone mineral density. Brazilian minimum wage per month = US$ 265.73.

### Body composition and biomarkers

No variables analyzed showed intergroup differences at T0 (*p* > 0,05). After supplementation there were changes in the results of body composition in the SG. In the CG, total body fat did not vary in relation to time 0 and time 60 (*p* = 0.639), and in the SG there was a decrease in the final median (*p* = 0.259). The percentage of total fat, android fat, and gynoid fat decreased significantly in the SG. The gynoid lean mass increased in both groups, however this increase was significant in the SG (*p* = 0.007). Intragroup bone mineral density did not change from time 0 to time 60 (CG, *p* = 0.233; SG, *p* = 0.433) (Table 2).

**Table 2:** Body composition in monozygotic twin adults with and without cholecalciferol supplementation.

	CG (n = 45)				SG (n = 45)				CG (T60) x SG (T60)	
	T0	T60	Effect size	*p* value	T0	T60	Effect size	*p* value	Effect size	*p* value
BMI, (kg/m^2^)	25.5 (21.3–30.5)	25.5 (20.5–30.1)	0.218	0.614	24.9 (22.1–33.9)	24.5 (21.6–33.8)	0.222	0.602	0.019	0.782
Total body fat, (kg)	18.1 (15.3–25.6)	18.1 (14.9–24.9)	0.054	0.639	19.2 (15.8–24.3)	18.6 (15.1–23.8)	0.116	0.259	0.042	0.812
Total body fat, (%)	29.9 (26.3–36.8)	30.7 (26.6–38.5)	0.048	0.758	32.3 (27.2–37.7)	30.5 (26.7–36.9)	0.120	0.001	0.013	0.977
Arm Fat, (%)	31.3 (25.9–38.0)	32.2 (26.1–39.5)	0.037	0.554	33.5 (27.8–40.8)	33.9 (27.3–40.3)	0.043	0.390	0.072	0.583
Trunk Fat, (%)	30.4 (24.9–36.4)	30.3 (25.1–36.4)	0.001	0.758	31.8 (25.7–38.4)	31.4 (27.7–36.9)	0.015	0.228	0.138	0.506
Android fat, (%)	29.8 (22.7–38.4)	27.2 (22.2–38.4)	0.074	0.360	31.7 (23.0–38.2)	29.8 (25.3–37.5)	0.050	0.001	0.113	0.548
Gynoid Fat, (%)	35.8 (29.5–42.3)	34.7 (29.6–43.4)	0.006	0.831	37.9 (30.8–43.0)	36.5 (29.8–41.5)	0.094	0.005	0.049	0.687
Leg Fat, (%)	33.5 (27.7–40.7)	32.7 (28.2–40.6)	0.030	0.097	35.4 (29.7–40.3)	35.0 (30.1–40.0)	0.001	0.692	0.122	0.480
Total Lean Mass, (kg)	39.0 (35.1–45.5)	38.6 (34.7–47.6)	0.091	0.839	38.2 (34.5–47.6)	38.2 (34.1–47.7)	0.015	0.554	0.067	0.781
Android Lean Mass, (kg)	26.2 (22.5–31.8)	26.3 (22.7–32.3)	0.017	0.906	26.2 (22.5–31.6	24.8 (22.3–33.7)	0.011	0.877	0.009	0.681
Gynoid Lean Mass, (kg)	61.1 (53.8–74.8)	62.0 (56.0–74.5)	0.005	0.616	58.3 (51.8–68.9)	59.6 (52.9–70.4)	0.052	0.007	0.193	0.362
BMC, (g)	2,248.5 (2060.7–2,558.5)	2,182.5 (2,309.0–2,542.8)	0.056	0.233	2,298.0 (2007.0–2,837.5)	2,197.0 (1950.5–2,976.8)	0.048	0.433	0.118	0.715

Continuous variables are shown as median (percentile 25–percentile 75) and compared by Wilcoxon test (intra-group values) and Mann–Whitney test (inter-group values). CG, Control group; SG, Supplemented group; T0, first analysis; T60, analysis 60 days after the first; BMI, body mass index; BMC, bone mineral density.

Three subjects in the CG presented deficiency in serum concentrations of 25 (OH) D at time 0, and after 60 days one additional subject presented insufficiency. The subject received guidance on the use of vitamin D supplementation after completion of the research. Regarding the SG, only two subjects were classified as deficiency in 25(OH) D at the beginning of supplementation (60 days), and at the end of supplementation, with the dose used in this study (2000 IU cholecalciferol), no subjects presented insufficient serum concentrations of 25 (OH) D. No subject presented potentially toxic concentrations of 25-hydroxy-vitamin D.

The concentration of 25 (OH) D in the serum increased in the SG in relation to the initial value (> 50 µg/d). Comparing the groups separately, the total cholesterol, triglycerides, ALT, uric acid, and creatinine demonstrated analytical and biological variation after 2 months, without pathologic significance. However, when evaluating the difference in biomarkers between the CG and SG, no significant variations were found, with the exception of 25 (OH) D (Table 3).

**Table 3:** Serum concentrations of biochemical variables in monozygotic twin adults with and without cholecalciferol supplementation.

	CG (n = 45)				SG (n = 45)				CG (T60) × SG (T60)	
	T0	T60	Effect size	*p* value	T0	T60	Effect size	*p* value	Effect size	*p* value
*25 (OH) D, ng/mL	32.7 (27.3–43.9)	34.4 (26.9–39.3)	0.050	0.382	30.7 (26.3–33.6)	50.6 (40.3–56.1)	1.763	< 0.001	1.422	< 0.001
Total proteins, g/dL	7.4 (6.9–8.1)	7.3 (6.5–7.9)	0.469	0.189	7.3 (7.0–8.1)	7.3 (6.5–7.7)	0.409	0.138	–	0.729*
Glucose, mg/dL	86.0 (79.0–97.0)	84.0 (79.0–93.0)	0.048	0.397	87.0 (79.0–94.5)	87.5 (82.0–91.0)	0.166	0.069	0.090	0.735
Total cholesterol, mg/dL	164.0 (143.0–188.0)	165.0 (144.0–182.0)	0.281	0.022	165.0 (138.5–185.5)	155.0 (129.0–173.5)	0.431	0.003	0.237	0.236
HDL cholesterol, mg/dL	38.0 (30.0–51.0)	45.0 (33.0–57.0)	0.414	0.076	38.0 (31.5–51.0)	41.0 (34.5–52.3)	0.349	0.080	0.026	0.886
Triglycerides, mg/dL	80.0 (64.5–118.5)	74.0 (57.0–92.0)	0.199	0.018	83.0 (65.5–118.0)	90.0 (66.8–114.8)	0.157	0.300	0.127	0.224
ALT, U/L	19.0 (12.5–31.0)	17.0 (12.0–22.0)	0.246	0.058	17.0 (11.5–31.0)	14.5 (11.0–20.8)	0.391	0.017	0.137	0.099
AST, U/L	20.0 (17.0–24.0)	19.0 (14.0–23.0)	0.184	0.314	19.0 (16.0–28.0)	18.5 (15.8–25.3)	0.372	0.070	0.098	0.477
Uric acid, mg/dL	4.0 (3.0–5.2)	3.7 (2.9–4.2)	0.312	0.007	4.0 (3.1–5.1)	3.6 (3.0–4.9)	0.362	0.003	0.024	0.985
Creatinine, mg/dL	0.9 (0.8–1.0)	0.8 (0.7–1.1)	0.349	0.031	0.9 (0.8–1.1)	0.8 (0.7–1.1)	0.278	0.039	0.034	0.856

Continuous variables are shown as median (percentile 25–percentile 75) and compared by Wilcoxon test (intra-group values) and Mann–Whitney test (inter-group values). CG, Control group; SG, Supplemented group; T0, first analysis; T60, analysis 60 days after the first; 25 (OH) D, 25-hydroxyvitamin D, reference value: > 20 ng/mL; Total proteins, reference value: 6.0–8.0 g/dL; Glucose, reference value: 70–99 mg/dL; Total cholesterol, reference value: < 200 mg/dL; HDL, high-density lipoprotein, reference value: > 60 mg/dL; Triglycerides, reference value: < 150 mg/dL; ALT, alanine aminotransferase, reference value: men 11–45 U/L, woman 10–37 U/L; AST, aspartate aminotransferase, reference value: men 11–39 U/L, woman 10–37 U/L; Uric acid, reference value: 1.0–7.0 mg/dL; Creatinine, reference value: men 0.70–1.20 mg/dL, woman 0.53–1.00 mg/dL.

*It was not possible to calculate the effect size (equals values).

### Gene expression

**Figure 2:**
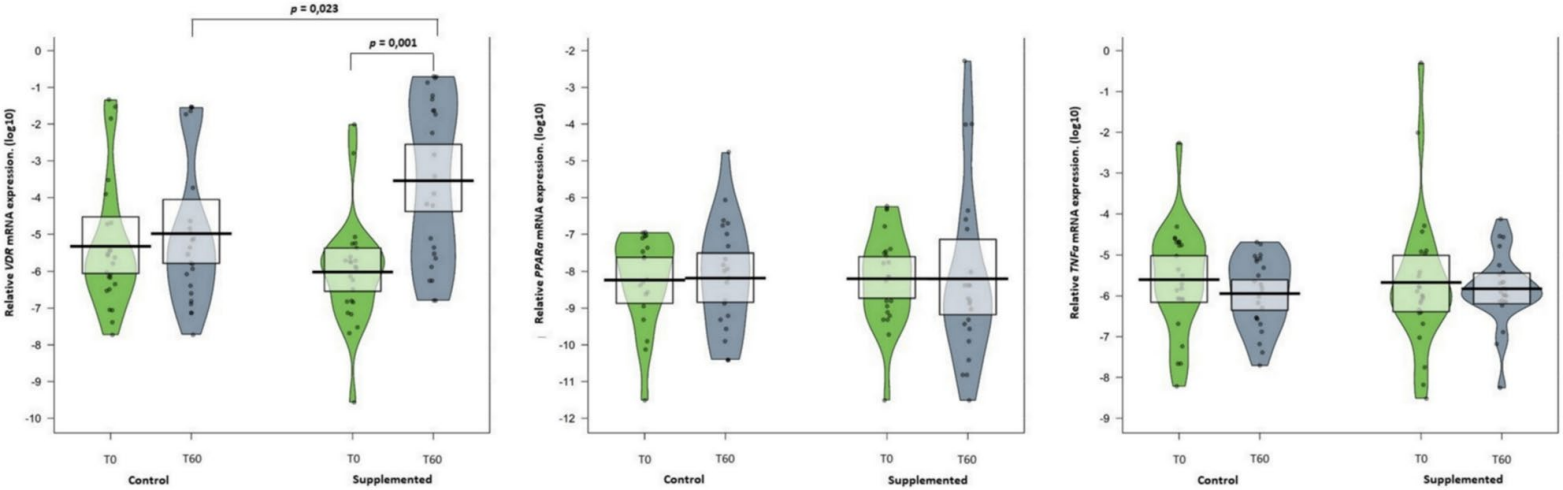
Median and distribution of VDR mRNA, TNFa mRNA, and PPARa mRNA expression in twins with and without supplementation with cholecalciferol. Data are shown as median (quartiles 1–3). Mann–Whitney U test was used to compare differences between independent groups (SG vs. CG). Wilcoxon test was used to compare intra-group values (T0 vs. T60). Vitamin D receptors (*VDR*); Tumor Necrosis Factor Alpha (*TNFa*); Peroxisome Proliferator Activated Receptor Alpha (*PPARa*).

The mRNA expression was performed in 50 MZ twins, 25 in the CG and 25 in the SG, as shown in (Fig. 2). Of the 3 genes analyzed in this study, mRNA expression of *VDR* increased significantly after 2 months of supplementation with 2000 IU cholecalciferol in the SG when compared with the CG (*p* = 0.001 and *p* = 0.023, respectively) (Supplementary Table 1 for sequence).

## Discussion

The findings of this short-term longitudinal study demonstrated that cholecalciferol supplementation at a concentration of 2000 IU increased circulating serum vitamin D levels and *VDR* mRNA expression, as well as decreasing the percentage of body fat in MZ. These results are innovative since, for the first time, the effect of an increase in vitamin D on mRNA expression and body composition was evaluated without genetic influence and the results collaborate with discussions about the ideal 25 (OH) D cut-off point to be considered, since concentrations above 50 ng/mL brought beneficial consequences to the individual's health.

The main objective of clinical research involving human beings is to improve prophylactic, diagnostic, and therapeutic procedures and to understand the etiology and pathogenesis of certain diseases. This studie provide us with greater understanding of these interactions, which may contribute to the development of strategies to combat diseases and promote health.

In Brazil, blood values of 25 (OH) D greater than 20 ng/mL are considered sufficient^22^, however, worldwide, there are several parameters considered as sufficient values, varying from > 10 ng/mL, > 30 ng/mL, and > 40 ng/mL^23^. These recommendations were established based on studies on bone health, and, to date, there are no recommendations that take into account other benefits that vitamin D may bring to the individual.

Circulating levels of 25 (OH) D correlate negatively with the body mass index and percentage of fat mass, since the higher the BMI and percentage of fat mass the lower the serum vitamin D concentration^11,24^. Obese healthy adults presented increased blood levels of 25 (OH) D after weight loss over a 12 month period^25^. In addition, although there is no clear evidence on the beneficial effect of vitamin D supplementation on metabolic parameters in obese individuals and weight loss^26^, our findings indicate a likely benefit for patients who need to decrease fat mass.

We present new evidence regarding the concentration of vitamin D and body composition, indicating that an increase in serum 25 (OH) D concentration and expression of the *VDR* gene without weight loss, since the BMI did not change during the period of 2 months, contributes to the reduction in body fat and a slight increase in gynoid lean mass. Therefore, studying its molecular bases, metabolic pathways and interaction with tissues, such as adipose tissue, is needed to assist in future practical and clinical actions for the control of diseases that affect a large part of the population^27^.

A study that supplemented vitamin D for 6 months at 2,000 IU/day in 40 overweight and obese adults found no alterations in BMI and body fat percentage; however the analysis was performed using bioimpedance^28^. We evaluated body composition with the reference standard for the assessment of body composition^29^. On the other hand, after 24 weeks of supplementation with 60,000 IU/week of cholecalciferol, subcutaneous truncal fat reduced in overweight and obese pre-diabetic Asian women, with vitamin D deficiency^1^.

Fuller and collaborators^30^ showed that adults over the age of 45 who received supplements to increase lean mass only gained more strength when vitamin D levels were above 30 ng/mL. After supplementation with 420 IU cholecalciferol/day for 12 months, men and women presented significantly increased lean body mass (from 43.8 ± 9.6 to 44.3 ± 9.8 kg)^31^.

These findings may be related to the fact that the vitamin D receptor is present in almost all human tissues and mediates the vitamin D biological actions in adipose tissue, where it can be activated or inactivated^32,33^. VDR is a transcription factor that plays an important role in inducing the expression of several genes involved in the pathogenesis of obesity^34^.

Vitamin D and its metabolites belong to a small set of dietary compounds that have direct effects on gene regulation^35^. *VDRs* are critical for vitamin D to perform its functions, since the active form of this micronutrient 1,25-dihydroxy-vitamin D (1,25(OH)2D), penetrates the cell membrane and binds to it. This complex interacts with the retinoic acid receptor (RXR) and forms heterodimers that act on target gene promoter regions, called vitamin D response elements, thus initiating a cascade of molecular interactions that regulate the transcription and suppression of specific genes^36^. *VDRs* have direct effects on the epigenome and expression of over 1,000 genes^37^. This gene and its ligands antagonize proinflammatory transcription factors, such as *NF-AT, AP-1*, and *NF-κB* in T cells, which results in decreased expression of cytokines such as *IL2* and *IL12*^38^.

A cohort study of 335 adults (age > 18 years old) with sickle cell anemia, followed at the Chicago Hospital in the United States, found that low vitamin D levels were associated with decreased *VDR* expression in peripheral blood mononuclear cells^39^. In white adipose tissue of obese rats, *VDR* gene expression increased after vitamin D supplementation of 50, 1,000, or 10,000 IU/kg for 12 weeks^40^. In 110 patients with oral neoplastic lesions, premalignant lesions, and oral cancer, *VDR* expression was increased, while insufficient or deficient blood levels of this micronutrient are prevalent in India^41^. On the other hand, a study on peripheral blood of 40 epileptic subjects found no significant correlation between vitamin D serum concentrations and *VDR* gene expression levels in Iran^42^. Tomaszewska, et al.^43^, after orally supplementing 16 patients with chronic rhinosinusitis with 1,000 IU of cholecalciferol for 1 month, in Warsaw, Poland, found no changes in *VDR* gene expression in sinonasal epithelial cells. These contradictory results indicate the importance of evaluating the effect of vitamin D supplementation in different population groups and on gene expression in different cell types and tissues.

Studies demonstrate that cholecalciferol can regulate gene expression by direct binding to VDR and that its affinity for VDR is several times higher than the active form of vitamin D (1,25-OH vitamin D) when bound to the vitamin D binding protein^44,45^. In humans, the Vitamin-D receptor (VDR) interacts with 1α,25(OH)2D3 in the cell nucleus to generate genomic responses. ChIP-chip, ChIP-seq analyses and FAIRE-qPCR analyses have shown that, less than 1,000 sites are generally occupied by the VDR in the absence of 1α,25(OH)2D3 and nearly 9,000 sites are changed following 24 h treatment with the vitamin-D. In effect stimulation with active vitamin-D increases the chromatin accessibility for VDR and its signalling^15,16,46^.

Studies with MZ are of the utmost importance, since each pair comes from a single zygote and they present genetically identical elements, i.e., have the same genotype and constitute the only isogenic series in the human species. In twin studies, to assess the importance of genotype in determining the phenotype, the following assumptions are accepted, 1. Twins are a sample of the general population, which is to say that among twins all characters are distributed in the same way as in the general population. 2. The elements of each pair of twins are subject to the same environmental influences. 3. The environment of the twins is, on average, equal to that of the elements of the general population. By accepting these assumptions, one also accepts that the quantitative characters are determined solely by environmental factors in MZ^14^. Each pair of twins in this research had the same conditions and lifestyle as their sibling.

The supplementation dose used by the MZ in the current study is the recommended daily dose to maintain adequate serum values in adults (2000 IU/day), unlike most studies on vitamin D supplementation, which have been performed at doses of 1,000 IU/day for a period of 12 months^47^, 2000 IU/day for 3 to 6 months^48,49^, 2,500 IU/day for 4 or 6 months^50,51^, 5,000 IU/day for 3 months^52^, 40,000 IU/weekly for a period of 2 months^53^, 50,000 IU/weekly in studies ranging from 5 to 6 months^54,55^, and 100,000 IU/monthly from 2 to 12 months^56,57^. A study developed with a maintenance dose of 2000 IU/day of vitamin D3 for 3 months in men and women who had been treated with 50,000 IU of vitamin D3 weekly for 3 months showed that this dose is not sufficient to maintain elevated levels of 25 (OH) D, however, serum values remained above 20 ng/mL^58^.

Experiments with vitamin D are aimed at population groups with 25(OH)D deficiency and some pathologies, such as diabetes, hypertension, insulin resistance, musculoskeletal disorders, cardiovascular disease, and kidney disease, as there is an association between low vitamin D levels and an increased risk of developing and worsening cardiovascular disease^59^, different types of cancers^60^, diabetes^61^, autoimmune diseases^62^, and overall mortality^63^. In addition, studies which used lower dosages of cholecalciferol were performed for periods longer than 2 months. For example, Lopez^64^ supplemented individuals with pre-diabetes with 2000 IU of cholecalciferol for 4 months and found a beneficial metabolic response, reducing circulating miR-7 and miR-192, accompanied by increased miR-152, which are useful biomarkers in prevention studies for diabetes.

Vitamin D has regulatory effects on innate and adaptive immunity and its receptor *VDR* is also expressed in cells of the immune system^65^. Therefore, the use of vitamin D3 and its analogues with other immunosuppressants has been studied synergistically or in isolation, in cases of autoimmune diseases, transplants, type 2 diabetes mellitus, and neoplasms^66,67^.

Pathogenic stimuli induce *TNFa* expression, which in turn induces other mediators and proteases responsible for the inflammatory response^68^. Omidian^69^, supplementing diabetic patients with 4,000 IU/day of vitamin D for 3 months, found a significant difference in serum *TNFa* levels, showing the possible protective effect that this micronutrient can have on inflammation when used in larger dosages and for a prolonged period. Borges^70^ showed an association between vitamin D deficiency and reduced beta-oxidation markers, including *PPARa*. However, in the current research no significant association was found between 2000 IU cholecalciferol supplementation with *TNFa* or *PPARa* mRNA expression in MZ, possibly owing to the healthy population used.

The major limitations of the present study were the use of a single concentration of the supplement and evaluations performed with only 2 months of use, which did not enable us to address the long-term effects of this supplementation. The small sample size, as our pilot study population consisted of same-sex MZ twin siblings and the decrease in the sample number for analysis of gene expression. However, this was the first clinical trial with vitamin D supplementation evaluating gene expression in this study population. Thus, further studies with different concentrations and different supplementation times are necessary to confirm our hypothesis that the increase in the serum concentration of vitamin D possibly contributes to the fight against cardiovascular diseases through the reduction in body fat.

In conclusion, increasing serum 25 (OH) D to values above 50 ng/mL yields health benefits through increased expression of *VDR* mRNA, reduced body fat, and increased lean mass, however, supplementation with 2000 IU of cholecalciferol for 2 months did not alter *TNFa* or *PPARa* mRNA expression in twins.

## Supplementary information

Supplementary Information 1.

Supplementary Information 2.

## Data Availability

All datasets generated for this study are available from the corresponding author on direct request.
