# Supplementary material for: The impact of vitamin D supplementation on VDR gene expression and body composition in monozygotic twins: randomized controlled trial
Source: Sci Rep. 2020 Jul 20;10:11943. doi: 10.1038/s41598-020-69128-2 (PMC7371728; doi:10.1038/s41598-020-69128-2)
Supplement: Supplementary file 1 — Supplementary Information 1. [file 41598_2020_69128_MOESM1_ESM.docx]

**The impact of vitamin D supplementation on VDR gene expression and body composition in monozygotic twins: randomized controlled trial**

**Authors**

Jeane Franco Pires Medeiros^1*^, Michelle Vasconcelos de Oliveira Borges^1^, Aline Alves Soares^1^, Jessica Cavalcante dos Santos^2^, Ana Beatriz Bezerra de Oliveira^2^, Conceição Horrana Belo da Costa^2^, Marina Sampaio Cruz^1^, Raul Hernandes Bortolin^4^, Renata Caroline Costa de Freitas^4^, Paulo Moreira Silva Dantas^1^, Mario Hiroyuki Hirata^4^, Vivian Nogueira Silbiger^3,5^, André Ducati Luchessi^1,3,5^

^1^Department of Health Sciences, Federal University of Rio Grande do Norte, Natal, RN, Brazil. jeanefpires@hotmail.com; vasmichelle@gmail.com;
aaline.alves@hotmail.com; marinasmcruz@gmail.com; pgdantas@icloud.com;
^2^Department of Pharmaceutical Sciences, Faculty of Pharmacy, Federal University of Rio Grande do Norte, Natal, RN, Brazil. c.jesk@hotmail.com;
anabboliveira@hotmail.com; horranabellocosta@hotmail.com;
^3^Graduate Program in Pharmaceutical Sciences, Faculty of Pharmacy, Federal
University of Rio Grande do Norte, Natal, RN, Brazil.
viviansilbiger@hotmail.com;
^4^Department of Clinical and Toxicological Analyses, School of Pharmaceutical
Sciences, University of Sao Paulo, Sao Paulo, SP, Brazil.
raulhbortolin@yahoo.com.br; renata_karoline@hotmail.com; mhhirata@usp.br;
^5^Department of Clinical and Toxicological Analyses, Federal University of Rio
Grande do Norte, Natal, RN, Brazil. andre.luchessi@outlook.com;

**SUPPLEMENTARY TABLE**

**Supplementary Table 1.** Gene expression in monozygotic twin siblings before and after supplementation with cholecalciferol.

|  | **CG (n = 25)** | | | | **SG (n = 25)** | | | | **CG (T60) x SG (T60)** | |
| --- | --- | --- | --- | --- | --- | --- | --- | --- | --- | --- |
|  | **T0** | **T60** | **Effect size** | ***p-value*** | **T0** | **T60** | **Effect size** | ***p-value*** | **Effect size** | ***p-value*** |
| ***VDR*** | 0.00242 (0.00152 - 0.00435) | 0.00304 (0.00152 - 0.00743) | 0.190 | 0.970 | 0.00321 (0.00111 - 0.00366) | 0.18599 (0.00602 - 0.41928) | 0.874 | 0.001 | 0.618 | 0.005 |
| ***PPARa*** | 0.00048 (0.00018 - 0.00081) | 0.00034 (0.00012 - 0.00079) | 0.251 | 0.132 | 0.00021 (0.00010 - 0.00057) | 0.00019 (0.00007 - 0.00137) | 0.294 | 0.730 | 0.079 | 0.436 |
| ***TNFa*** | 0.00331 (0.00223 - 0.0091) | 0.00300 (0.00160 - 0.00610) | 0.242 | 0.126 | 0.00261 (0.00177 - 0.00444) | 0.00340 (0.00210 - 0.00830) | 0.262 | 0.679 | - | 0.860* |

Continuous variables are shown as median (percentile 25 - percentile 75) and compared by Wilcoxon test (intra-group values) and Mann-Whitney test (inter-group values). CG, Control group; SG, Supplemented group; T0, first analysis; T60, analysis 60 days after the first; *VDR*, Vitamin D Receptor Proteins; *PPARa*, Peroxisome Proliferator Activated Receptor Alpha; *TNFa*, Tumor Necrosis Factor Alpha.

* It was not possible to calculate the effect size (equals values).
