# Supplementary material for: The impact of vitamin D supplementation on VDR gene expression and body composition in monozygotic twins: randomized controlled trial
Source: Sci Rep. 2020 Jul 20;10:11943. doi: 10.1038/s41598-020-69128-2 (PMC7371728; doi:10.1038/s41598-020-69128-2)
Supplement: Supplementary file 2 — Supplementary Information 2. [file 41598_2020_69128_MOESM2_ESM.docx]

**The impact of vitamin D supplementation on VDR gene expression and body composition in monozygotic twins: randomized controlled trial**

**Authors**

Jeane Franco Pires Medeiros^1*^, Michelle Vasconcelos de Oliveira Borges^1^, Aline Alves Soares^1^, Jessica Cavalcante dos Santos^2^, Ana Beatriz Bezerra de Oliveira^2^, Conceição Horrana Belo da Costa^2^, Marina Sampaio Cruz^1^, Raul Hernandes Bortolin^4^, Renata Caroline Costa de Freitas^4^, Paulo Moreira Silva Dantas^1^, Mario Hiroyuki Hirata^4^, Vivian Nogueira Silbiger^3,5^, André Ducati Luchessi^1,3,5^

^1^Department of Health Sciences, Federal University of Rio Grande do Norte, Natal, RN, Brazil. jeanefpires@hotmail.com; vasmichelle@gmail.com;
aaline.alves@hotmail.com; marinasmcruz@gmail.com; pgdantas@icloud.com;
^2^Department of Pharmaceutical Sciences, Faculty of Pharmacy, Federal University of Rio Grande do Norte, Natal, RN, Brazil. c.jesk@hotmail.com;
anabboliveira@hotmail.com; horranabellocosta@hotmail.com;
^3^Graduate Program in Pharmaceutical Sciences, Faculty of Pharmacy, Federal
University of Rio Grande do Norte, Natal, RN, Brazil.
viviansilbiger@hotmail.com;
^4^Department of Clinical and Toxicological Analyses, School of Pharmaceutical
Sciences, University of Sao Paulo, Sao Paulo, SP, Brazil.
raulhbortolin@yahoo.com.br; renata_karoline@hotmail.com; mhhirata@usp.br;
^5^Department of Clinical and Toxicological Analyses, Federal University of Rio
Grande do Norte, Natal, RN, Brazil. andre.luchessi@outlook.com;

**SUPPLEMENTARY TABLE**

**Supplementary Table 2.** Primer sequences for mRNA quantification by qPCR.

| **Gene** | **Primer sequences** |
| --- | --- |
| ***VDR*** | forward: 5´ CTGGAGACTTTGACCGGAAC 3´  reverse: 5´ GTCCACACAGCGTTTGAGC 3´ |
| ***PPARA*** | forward: 5´ CAATGCACTGGAACTGGATG 3´  reverse: 5´ GAGAAAGATATCGTCCGGGT 3´ |
| ***TNFa*** | forward: 5´ GACACCATGAGCACTGAAAGC 3´  reverse: 5´ GCCAGAGGGCTGATTAGAGA 3´ |
| ***ACTB*** | forward: 5´ TTCTACAATGAGCTGCGTGTG 3´  reverse: 5´ ATAGCACAGCCTGGATAGCAA 3´ |
| ***GAPDH*** | forward: 5´ GCTGAGTACGTCGTGGAGTC 3´  reverse: 5´ CTGATGATCTTGAGGCTGTTG 3´ |
| ***18S rDNA*** | forward: 5′ CGCTACTACCGATTGGATGG 3′  reverse: 5′ AGTTCGACCGTCTTCTCAGC 3′ |
